# Novel role of immune-related non-coding RNAs as potential biomarkers regulating tumour immunoresponse via MICA/NKG2D pathway

**DOI:** 10.1186/s40364-023-00530-4

**Published:** 2023-10-02

**Authors:** Jing Zhang, Qizhi Luo, Xin Li, Junshuang Guo, Quan Zhu, Xiaofang Lu, Leiyan Wei, Zhiqing Xiang, Manqing Peng, Chunlin Ou, Yizhou Zou

**Affiliations:** 1 Department of Pathology, Xiangya Hospital, Central South University, Changsha, 410008 Hunan China; 2 Department of Immunology, School of Basic Medicine, Central South University, Changsha, 410000 Hunan China; 3 National Clinical Research Center for Geriatric Disorders, Xiangya Hospital, Central South University, Changsha, 410000 Hunan China

**Keywords:** MICA, NKG2D, circRNA, lncRNA, microRNA, ceRNA, Immunity, Cancers

## Abstract

Major histocompatibility complex class I related chain A (MICA) is an important and stress-induced ligand of the natural killer group 2 member D receptor (NKG2D) that is expressed in various tumour cells. Given that the MICA/NKG2D signalling system is critically embedded in the innate and adaptive immune responses, it is particularly involved in the surveillance of cancer and viral infections. Emerging evidence has revealed the important roles of non-coding RNAs (ncRNAs) including microRNAs (miRNAs), long noncoding RNAs (lncRNAs) and circular RNAs (circRNAs) in different cancer types. We searched for all relevant publications in the PubMed, Scopus and Web of Science database using the keywords ncRNA, MICA, NKG2D, cancer, and miRNAs. All relevant studies published from 2008 to the 2023 were retrieved and collated. Notably, we found that miRNAs can target to NKG2D mRNA and MICA mRNA 3’-untranslated regions (3’-UTR), leading to translation inhibition of NKG2D and MICA degradation. Several immune-related MICA/NKG2D pathways may be dysregulated in cancer with aberrant miRNA expressions. At the same time, the competitive endogenous RNA (ceRNA) hypothesis holds that circRNAs, lncRNAs, and mRNAs induce an abnormal MICA expression by directly targeting downstream miRNAs to mediate mRNA suppression in cancer. This review summarizes the novel mechanism of immune escape in the ncRNA-related MICA/NKG2D pathway mediated by NK cells and cancer cells. Moreover, we identified the miRNA-NKG2D, miRNA-MICA and circRNA/lncRNA/mRNA-miRNA-mRNA/MICA axis. Thus, we were particularly concerned with the regulation of mediated immune escape in the MICA/NKG2D pathway by ncRNAs as potential therapeutic targets and diagnostic biomarkers of immunity and cancer.

## Introduction

Immune checkpoint inhibitors (ICIs) represent new approaches for durable immune control in cancers. The identification of anti-tumour therapies that combat immune escape is a significant challenge [1]. Natural killer (NK) cells are innate cytotoxic lymphoid cells that are essential for tumour cell elimination and cancer immunosurveillance [2]. NK cells are at the forefront of many immunotherapeutic strategies that produce a variety of cytokines and chemokines [3]. NK cell activation is influenced by the strong regulation of activating and inhibitory signals generated by the engagement of diverse receptors. NK cells can kill aberrant cells, including tumour and virus-infected cells. This is because of the high expression of its activated receptor, natural killer group 2 member D receptor (NKG2D), also known as killer cell lectin-like receptor K1 (KLRK1), after receiving the ligand signal [4]. NKG2D located on human chromosome 12p13 and is a C-type lectin like activating receptor expressed in NK cells, γδ T cells, CD8^+^ T cells, and some auto-reactive or immunosuppressive CD4^+^ T cells. It is an important recognition receptor for the detection and elimination of virus-infected cells and various cancer cells [4].

NKG2D is an immune checkpoint target that may also be a potential ICI as a receptor for NK cell activation [5]. In humans, NKG2D recognizes 8 different NKG2D ligands (NKG2DLs), including major histocompatibility complex class I chain A (MICA) and MICB. The remaining NKG2DLs are members of the UL-16 binding protein (ULBP) family, also known as retinoic acid early transcripts (RAET) 1. Therefore, they have been named ULBP-1 (RAET1I), ULBP-2 (RAET1H), ULBP-3 (RAET1N), ULBP-4 (RAET1E), ULBP-5 (RAET1G) and ULBP-6 (RAET1L) [1]. By binding to the activator receptor NKG2D on the NK and T cell subpopulations, these ligands trigger immune efflux responses, including cytolysis and cytokine production, through costimulation of the primary activation of responding lymphocytes or cells that receive signals via the T cell antigen receptor (TCR). NKG2D ligand dysregulation may be a major source of immunogenicity in dysregulated cells. Hence, understanding the regulatory mechanisms of NKG2D ligands is important, as their expression may be a key factor in determining whether a cell is visible to the immune system [1].

NKG2D regulation and lymphocyte stress monitoring are particularly relevant in cancer immunotherapy [3, 6]. The gene encoding MICA is located on human chromosome 6p21.31. The map of the MICA gene within the major histocompatibility complex is highly polymorphic and the alleles are expressed in a codominant manner [7]. The MICA protein encoded by most of the alleles is composed of three extracellular domains, a transmembrane domain, and a cytoplasmic tail [8]. NKG2D binding to MICA in target cells triggers the catalytic activation of NK cells, resulting in target cell lysis via perforin release by the effector cells [9]. MICA stimulation of NK cells leads to strong activation and tumour cell rejection with enhanced NKG2D-mediated cytotoxicity [9, 10]. These concepts have further increased interest in exploiting the immunotherapeutic potential of MICA-NKG2D axis dependent NK cells for the treatment of cancer in cancer [9, 11].

MICA undergoes post-translational modifications that regulate their expression as they are called membrane-bound MICA (mMICA) at the cell surface. However, whether MICA is also expressed on the surface of normal cells remains controversial. Any cell or cancer type can express MICA if appropriately stimulated [1, 3]. MICA expression was recently reported in many tumours and normal epithelial cells, but with predominantly intracellular localization and low cell surface expression. In addition, MICA upregulation is often associated with osmotic and oxidative stress, viral infections, and increased cell proliferation, which cannot be explained by DNA damage. Hence, MICA overexpression may be a valid strategy to limit tumour progression, as tumours exhibit escape strategies that subvert the biological functions of NKG2D [12]. Furthermore, the proteolytic cleavage of MICA by adisintegrin metalloproteinase domains (ADAMs) and tumour secreted metalloproteases (MMPs) or secretion in exosomes is the underlying mechanism [13]. NK cells recognise MICA on the tumour cell surface through NKG2D and promote a cytotoxic response leading to tumour cell elimination. At the same time, the expression levels of MICA on the tumour cell surface may determine anti-tumour efficacy, while those shed in the serum may act as a decoy of NKG2D to avoid immune rejection. The released soluble MICA (sMICA) can bind to NKG2D and induce the downregulation and degradation of NKG2D in NK and T cells, reducing NKG2D-dependent effector functions, resulting in decreased cytotoxicity of these immune effector cells and facilitating tumour immune evasion [4, 14, 15]. Consequently, increased levels of soluble MICA (sMICA), decreased mMICA in malignant cells, and decreased NKG2D in NK and effector T cells represent important aspects of immune escape [4]. Therefore, the regulation of human NKG2D ligand expression requires further investigation.

Many viruses interfere with NK cell activating ligands to limit cell activation, thereby reducing NK-mediated surveillance. According to these reports, MICA is associated with viral infections, including human cytomegalovirus (HCMV) [16], Epstein-Barr virus (EBV) [17], hepatitis B virus (HBV) [18], and severe acute respiratory syndrome corona virus 2 (SARS-CoV-2) [16, 17, 19]. For example, Corona virus disease 2019 (COVID-19), caused by severe acute respiratory SARS-CoV-2, has spread worldwide, using the respiratory tract as the main invasion site to cause acute respiratory diseases with fever, cough, and shortness of breath as the main symptoms [19]. SARS-CoV-2 inhibits the synthesis of several NK cell ligands, including MICA, and reduces NK cell activation [19]. In addition, the mMICA level decreased in patients with SARS-CoV-2, reducing the hosts’ ability to kill infected cells as well as their cytotoxic capacity [20]. SARS-CoV-2 infection generates more sMICA through the overexpression of ADAM17 metalloproteinase after the interaction between ACE2 and spike proteins, which reduces NK cell activation [21].

In the past decade, the vital roles of non-coding RNAs (ncRNAs) including circRNAs, long noncoding RNAs (lncRNAs) and microRNAs (miRNAs) also have significant functions in cancer [22, 23]. An increasing number of studies focusing on the relationship between ncRNAs and human diseases have been conducted in the context of the widespread dysregulation of ncRNA expression and function in human cancers [24–26]. The dysregulation of these ncRNAs is closely related to tumourigenesis [22, 27]. Emerging evidence indicates that miRNAs are involved in tumourigenesis, cell cycle control, metabolism, apoptosis, and tumour progression [28]. Although MICA can be shed at the surface of cancer cells during tumour development and progression, miRNA regulation is another important mechanism that disrupts MICA expression. Emerging data suggest that miRNAs are often aberrantly expressed in cancer and regulate key genes involved in tumour proliferation and metastasis.

A competitive endogenous RNA (ceRNA) hypothesis was recently proposed. RNAs can reportedly crosstalk with common miRNAs [29]. According to this hypothesis, lncRNA, circRNA and mRNA function as ceRNAs by regulating the post-transcriptional expression of genes that acting as miRNA sponges [30]. Unbalanced ceRNA networks resulting from lncRNA/circRNA/mRNA-miRNA-mRNA axis interactions contribute to cancer development [25]. Furthermore, abnormal gene expression alters the stability of ceRNA networks, highlighting the role of molecular biomarkers in optimising cancer treatment and management. Aberrant ceRNA activity may lead to diseases, even in the occurrence of cancer. Therefore, the development of prognostic signatures based on cancer-specific ceRNA networks is important for predicting clinical outcomes [30]. In summary, these results suggest that the cancer-specific ceRNA network involved in MICA-NKG2D pathway regulation could contribute to carcinogenesis and immune evasion.

This review guides the reader through the immunological aspects, emphasizing the roles of ncRNAs as modulators of the immune response mediated by the MICA-NKG2D pathway. In cancer, ncRNAs targeting the immune system may represent a promising approach for future immune treatments.

### Dysregulation of MICA/NKG2D expression in human tumuors

**Fig. 1:**
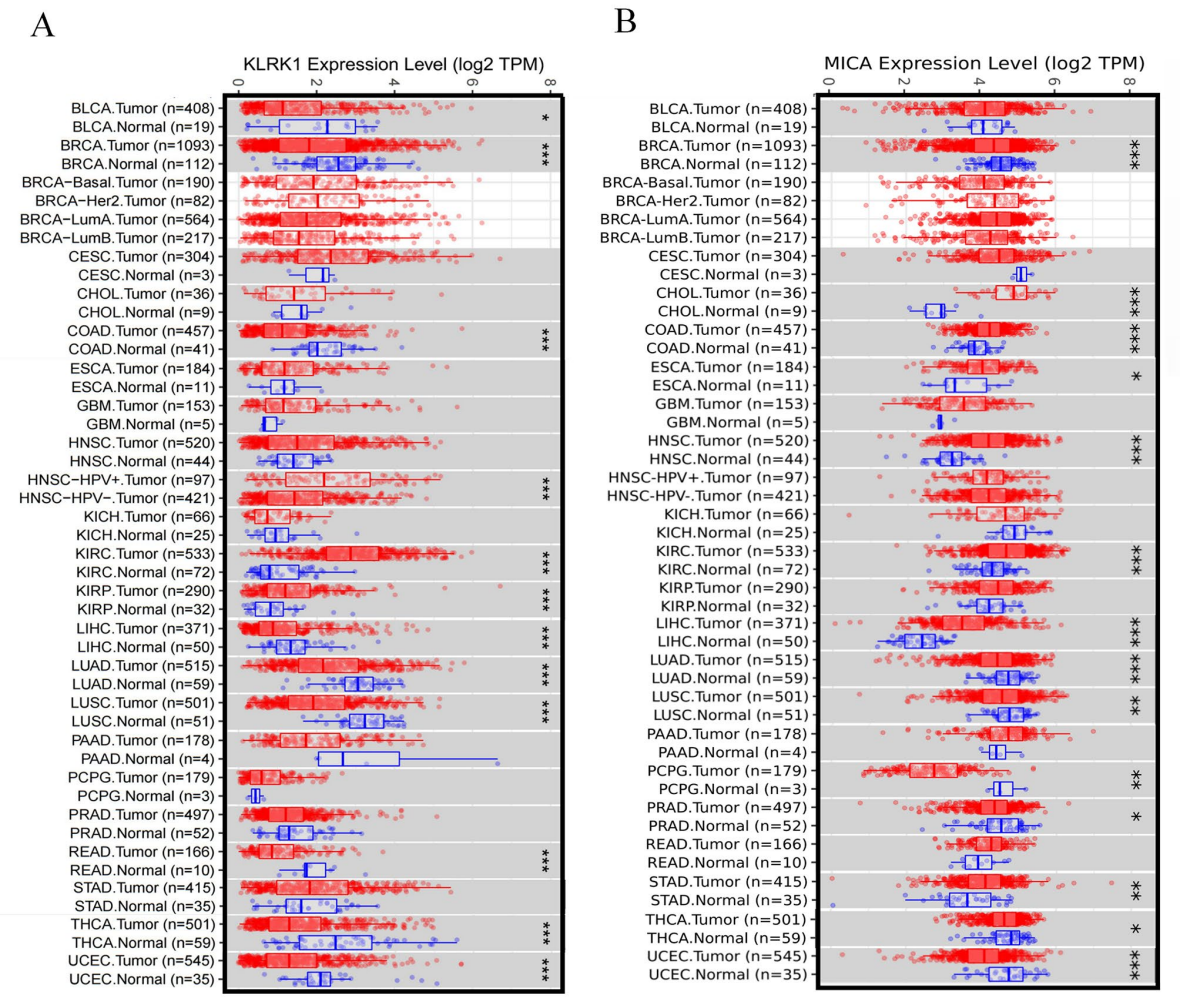
(**A**). The expressions of natural killer group 2 member D receptor (NKG2D), also known as killer cell lectin-like receptor K1 (KLRK1) and (**B**). MICA in human cancers. The expressions of NKG2D (KLRK1) and MICA in different human cancer types were evaluated by analysis of the RNA-seq data from The Cancer Genome Atlas database using the TIMER 2.0 program. *P < 0.05; **P < 0.01; ***P < 0.001

Aberrant NKG2D expression in different human cancer types was evaluated in the TIMER 2.0 program by analysing the RNA sequence data from The Cancer Genome Atlas (TCGA) data (http://timer.cistrome.org/) (Fig. 1A). Computational and biological evidence has indicated significantly different NKG2D expression in normal and cancerous tissues. Notably, NKG2D expression is downregulated in bladder urothelial carcinoma (BLCA), breast invasive carcinoma (BRCA), colon adenocarcinoma (COAD), liver hepatocellular carcinoma (LIHC), lung adenocarcinoma (LUAD), lung squamous cell carcinoma (LUSC), rectum adenocarcinoma (READ), thyroid carcinoma (THCA), uterine corpus endometrial carcinoma (UCEC). Conversely, NKG2D expression is upregulated in kidney renal clear cell carcinoma (KIRC) and kidney renal papillary cell carcinoma (KIRP). The patients showed higher NKG2D expression levels in head and neck squamous cell carcinoma (HNSC) when they were infected with human papillomavirus (HPV).

MICA is a stress-induced ligand of the NK cell activating receptor NKG2D, which is essential for NK cells to kill virus-infected cells and tumour cells [1]. Meanwhile, the aberrant expression of MICA in normal tissues and cancer tissues was also evaluated using TIMER 2.0 program by analysing RNA sequence data from TCGA data (Fig. 1B). Notably, MICA expression is downregulated in breast invasive carcinoma (BRCA), lung adenocarcinoma (LUAD), lung squamous cell carcinoma (LUSC), pheochromocytoma and paraganglioma (PCPG), prostate adenocarcinoma (PRAD), thyroid carcinoma (THCA) and uterine corpus endometrial carcinoma (UCEC). Patients showed higher levels of MICA expression in cholangiocarcinoma (CHOL), colon adenocarcinoma (COAD), esophageal carcinoma (ESCA), head and neck squamous cell carcinoma (HNSC), kidney renal clear cell carcinoma (KIRC), liver hepatocellular carcinoma (LIHC), stomach adenocarcinoma (STAD).

### Regulation of MICA/ NKG2D by miRNA to influence immune response

**Fig. 2:**
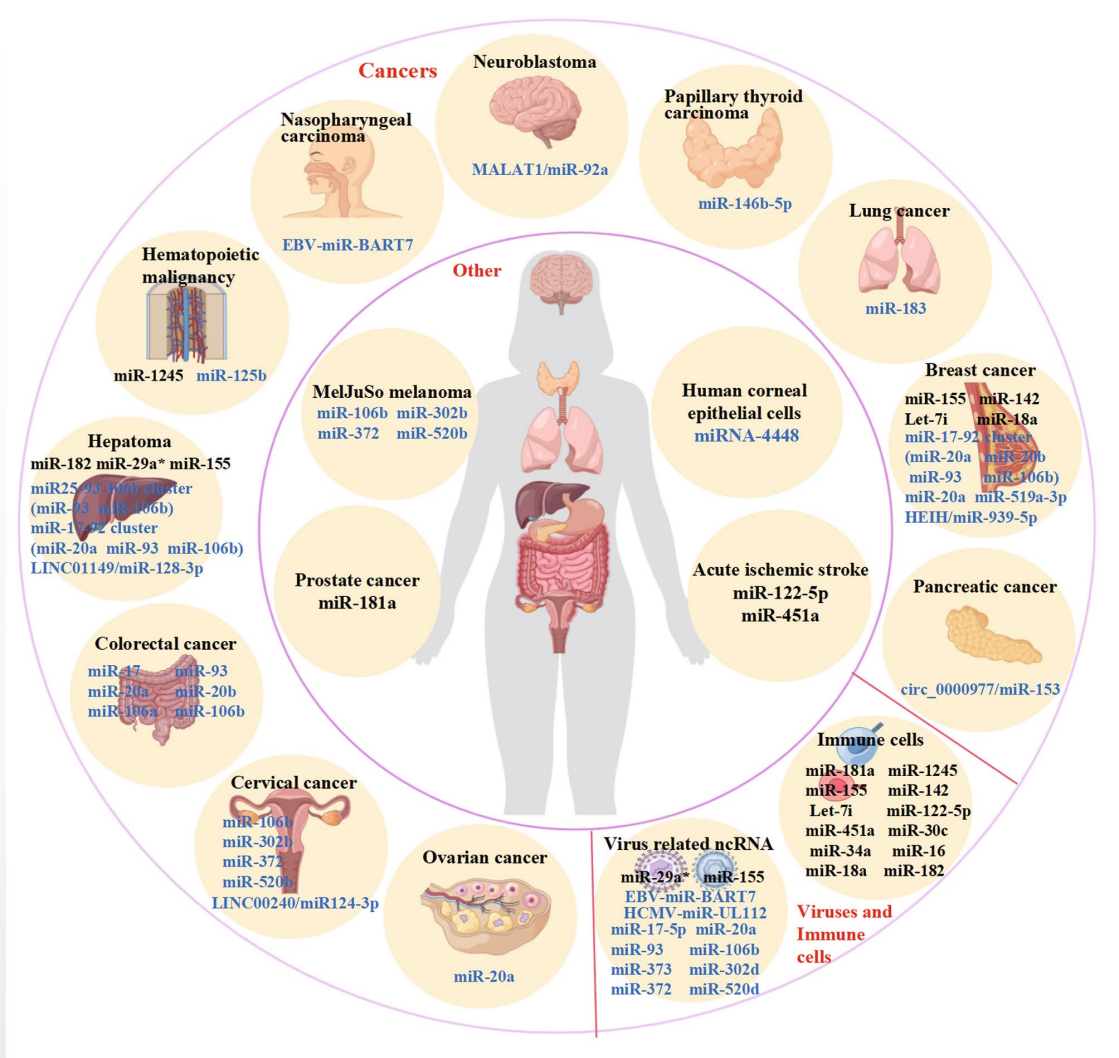
Non-coding RNAs in various cancers and cells play important roles in regulating NKG2D (black) and MICA (blue)

**Fig. 3:**
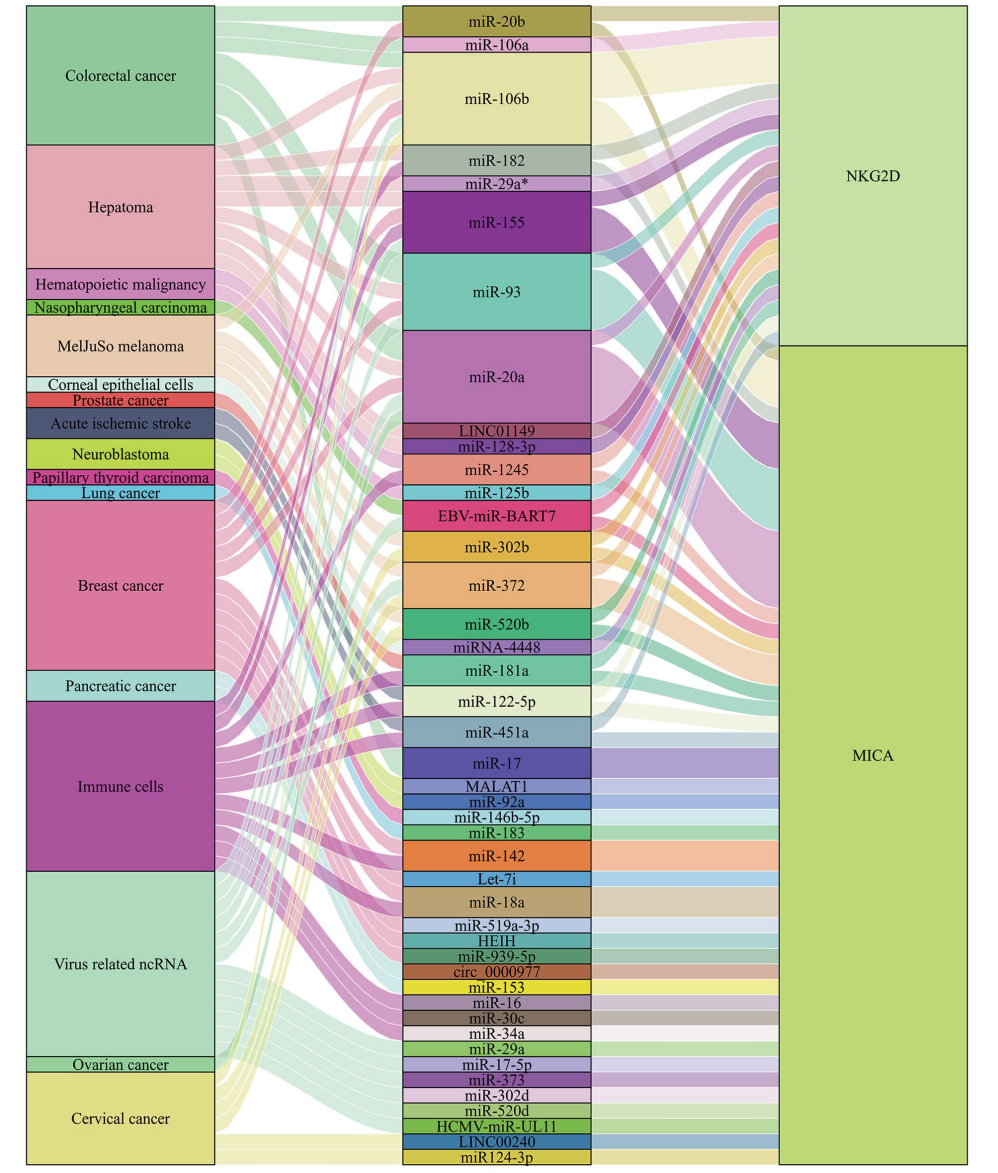
Sankey diagram showing that MICA/NKG2D pathway can be targeted by a variety of ncRNAs in various cancers and cell types

These MICA/ NKG2D associated miRNAs provide insight into the development of novel therapeutic cancer targeting medicines (Figs. 2 and 3; Table 1). The significantly decreased NKG2D expression and lower number of γδ T cells were detected in the peripheral blood of patients with prostate cancer. On the contrary, miR-181a increases in peripheral γδ T cells from prostate cancer patients. The inverse correlation was revealed between miR-181a and NKG2D expression in γδ T cells isolated from the thymus and peripheral blood. This strategy phenocopied miR-181a overexpression, which negatively regulated NKG2D expression in peripheral blood T cells at both the mRNA and protein levels, with the consequence of pathophysiological implications in cancer immunity and inflammatory disease settings. MiR-181a impaired the differentiation of human γδ T cells into tumour necrosis factor-α (TNF-α) associated effector T cells and decreased NKG2D expression [31].

**Table 1:** Regulation of MICA/ NKG2D by miRNA to influence immune response

Targeted immunity genes	MicroRNAs	Major outcomes	Related Disease/cell Types	References
NKG2D (KLRK1)	miR-181a	miR-181a impaired the differentiation of human γδ T cells into TNF-α associated effector T cells and decreased the expression of NKG2D.	prostate cancer/γδ T cells	[31]
	miR-1245	miR-1245 induced by TGF-β1, targets and downregulates NKG2D expression in NK cells, impairs NK cell functions and immune responses.	NonHodgkin’s lymphoma/Acute myelogenous leukemia/NK cells	[32]
	miR-155 miR-142 Let-7i	Tumour-derived exosomes (tex) with tex-miR-155 enhanced but tex-miR-142 and tex-Let-7i downregulated NKG2D mRNA expression. They induced appropriate immune response maturation by enhancing dendritic cells and impacted on immune suppressed tumour microenvironment.	breast cancer/dendritic cells	[33, 34]
	miR-122-5p miR-451a	Inhibition of miRNA-451a or miRNA-122-5p significantly increased the expression of NKG2D associated with activation in NK cells and may affect the function of circulating NK cells after acute ischemic stroke.	acute ischemic stroke/NK cells	[35]
	miR-30c	miR-30c increased NKG2D protein expression on the surface of NKL cells, enhanced the cytotoxicity.	NKL cells	[36]
	miR-34a	miR-34a targets NKG2D as a major hub in the T cell regulatory network, suggesting that miR-34a acts as an intervention target to regulate T cell immune responses in a wide range of tumour contexts	T cells	[37]
	miR-16	miR-16 targeted NKG2D, overexpression of miR-16 inhibited NKG2D expression in CD8^+^ T cells. Especially in miR-15-/16- mice, CD8^+^NKG2D^+^ T cells were increased, activation and cytotoxicity of T cells.	T cells	[38]
	miR-18a	miR-18a downregulates NKG2D, and impairment of NK cell cytotoxicity.	breast cancer/NK cells	[39]
	miR-182	miR-182 was significantly increased in NK cells from HCV-infected patients, while NKG2D mRNA expression was decreased, and overexpression of miR-182 reduced NKG2D mRNA expression. MiR182 mRNA was positively correlated with NKG2D mRNA in HCC.	NK cells from HCV-infected patients/HCV liver tissues/HCV infected cells/hepatocellular carcinoma	[40, 41]
	miR-29a* miR-155	miR-29a* and miR-155 decreased NKG2D mRNA expression, resulting in a decrease in NK cell lysis function accompanied by an 80% increase in viral load.	NK cells of HCV patients/HCV Huh cell lines	[42]
MICA	HCMV-miR-UL112	HCMV-miR-UL112 may also have binding sites with human cellular miRNAs to regulate MICA expression under certain conditions.	\	[43]
	miR-17-5p miR-20a miR-93 miR-106b miR-373 miR-302d miR-372 miR-520d	Hcmv-miR-UL112 may also have binding sites with human cellular miRNAs (miR-17-5p, miR-20a, miR-93, miR-106b, miR-373 and miR-520d) resulted in down-regulation of MICA and decreased susceptibility of NK cells due to avoid NKG2D-mediated MICA immune recognition in vitro and in vivo. miR-302d and miR-372 no effect on MICA in HeLa cells and DU145 cells.	Hcmv-miR-UL112 infected cells/HeLa cells/DU145 cells/primary human foreskin fibroblast (HFF) cells/human umbilical vein endothelial cells (HUVECs)	[43]
	miR-106b miR-302b miR-372 miR-520b	miR-106b, miR-302b, miR-372 and miR-520b target to MICA 3’-UTR region and dramatically downregulate MICA protein expression. miR-520b was induced by IFN-γ that could be sufficient to reduce the MICA protein and transcript expression.	MelJuSo melanoma and HeLa cell lines	[44]
	miRNA-4448	IFN-γ enhances the expression of MICA mRNA and protein levels by down-regulating miRNA-4448, thereby enhancing cytotoxicity and HCEC apoptosis mediated by NK and CD8^+^ T cells on corneal epithelium.	human corneal epithelial cells	[45, 46]
	miR-146b-5p	miR-146b-5p target the 3’-UTR of MICA; down-regulation of miR-146b-5p leads to increased expression of MICA in PTC cells and also to increased expression of NKG2D in cancer cells; inhibition of the MICA-NKG2D axis by miR-146b-5p may be one of the ways that PTC cells help tumours evasion immune response.	papillary thyroid carcinoma	[50]
	EBV-miR-BART7	EBV-miR-BART7 suppresses MICA mRNA and protein expression, reducing the sensitivity of NPC cells to NK92MI cells.	nasopharyngeal carcinoma/HK1 cells	[53]
	miR-125b	miR-125b as a tumour suppressor is commonly deregulated in cancer that its overexpression downregulated c-Myc mRNA and IRF4 mRNA but upregulated MICA mRNA and protein expression.	multiple myeloma	[57]
	miR-20a	miR-20a downregulated MICA expression, reduced NK cell cytotoxicity, and was NKG2D dependent in cancer cells to promote tumour growth.	colorectal cancer	[60, 61]
	miR-20a	The regulation of MICA by miR-20a in ovarian cancer is similar to that in colorectal cancer.	ovarian cancer	[62]
	miR-17-92 cluster (miR-20a miR-20b miR-93 miR-106b)	The regulation of MICA by miR-17-92 cluster in breast cancer is similar to that in ovarian and colorectal cancer. Suberoylanilide hydroxamic acid (SAHA) and valproic acid (VPA) treatment upregulated the expression of the MICA and typically decreased in the expression of the miR-17-92 cluster, increasing the sensitive of NK cell mediated cytotoxicity.	breast cancer	[64]
	miR-20a	3’-O-acetylvitexin effected cytotoxic and successfully increased MICA expression that had impact on miR-20a validated targets MICA.	triple-negative breast cancer	[67]
	miR-519a-3p	MiR-519a-3p binds to the MICA 3’-UTR and reduces the MICA mRNA levels and surface protein levels, impairs NK cell-mediated killing of BC cells and deregulates apoptosis by downregulating MICA.	breast cancer	[66]
	MiR-25-93-106b cluster (miR-93 miR-106b)	MiR-25-93-106b cluster can regulate sensitivity to NK cells, tumor progression and invasion by regulating the expression level of MICA.	hepatocellular carcinoma	[70]
	miR-17-92 cluster (miR-20a miR-93 miR-106b)	HDACi promoted the protein expression of MICA by inhibition of the transcription of MICA-targeting miRNAs (miR-20a, miR-93, and miR-106b) in HCC.	hepatocellular carcinoma	[71]
	miR-183	Knockdown TGF-β mRNA effectively resulting miR-183 expression was reduced but MICA protein expression was up-regulated. Silencing miR-183 and overexpression MICA contributed to the lysis of tumour cells by activated CD8^+^ T cells via the MICA-NKG2D pathway.	lung cancer	[74]

Moreover, miR-1245 was abundant in the exosomes of seven patients with non-Hodgkin’s lymphoma patients and three patients with acute myeloid leukaemia/myelodysplastic syndrome patients. MiR-1245 has target sites in the 3’-untranslated regions (3’-UTR) region of NKG2D mRNA and downregulates NKG2D expression in NK cells. The overexpression of mature miR-1245 in NK cells resulted in a sustained downregulation of the NKG2D receptor, leading to the escape of NK cell-mediated killing, therefore, miR-1245 is a negative regulator of NKG2D expression. MiR-1245 knockdown increased NKG2D protein and transcript levels. Transforming growth factor–β1 (TGF-β1) acts as a potent suppressor of NKG2D in NK cells, by enhancing the expression of mature miR-1245 post-transcriptional regulation and leading to impairing NK cell functions and immune responses due to NKG2D downregulation [32].

NKG2D was down-regulated by miR-142 and Let-7i, but up-regulated by miR-155 in exosomes isolated from a mouse mammalian breast cancer (BC) cell line. MiR-142 and Let-7i downregulation of NKG2D regulated dendritic cells (DCs) maturation and affected the tumour microenvironment through different mechanisms [33, 34]. One study reported that miR-155 directly enhanced NKG2D, but had little effect on DC maturation [33].

Brain ischaemia significantly and transiently suppresses NK cell number and activity of in the peripheral blood [35]. NKG2D expression in NK cells was also reportedly reduced during the acute phase but recovered at subsequent time points. However, miR-122-5p and miR-451a were significantly upregulated in human peripheral blood NK cells and damaged NK cells following acute ischaemic stroke. The inhibition of miRNA-451a or miRNA-122-5p significantly increased NKG2D expression, which is associated with NK cell activation and may affect circulating NK cell function after acute ischaemic stroke [35].

Overexpression of miR-30c reportedly increases NKG2D protein expression on the surfaces of NKL cells and enhances their cytotoxicity, whereas miR-30c inhibition has the opposite effect [36].

MiR-34a targets NKG2D as a major hub in the T cell regulatory network, suggesting that it acts as an intervention target to regulate T cell immune responses in a wide range of tumours [37].

MiR-16 targets the NKG2D 3’-UTR region, and its overexpression inhibits NKG2D expression in CD8^+^ T cells. Particularly in miR-15^−^/16^−^ mice, CD8^+^NKG2D^+^ T cells are increased, while T cells become activated and cytotoxic [38].

MiR-18a belongs to the miR-17-92 cluster, which targets the 3’-UTR of NKG2D and can downregulate NKG2D expression and impair NK cell cytotoxicity in mice with BC. In many patients with BC, indoleamine-2, 3-dioxygenase 1 (IDO1) mRNA and miR-18a expressions are elevated, whereas NKG2D mRNA expression is decreased. IDO1 impairs NK cell function mediated by miR-18a. This is a novel functional pathway for IDO1 that counteracts the immune responses in cancer [39].

MiR-182 plays contradictory roles in NK cells and hepatocytes during hepatitis C virus (HCV) infection [40]. The relative expression of miR-182 is significantly increased in NK cells from HCV-infected patients, while NKG2D mRNA expression is decreased, and miR-182 overexpression reduces NKG2D mRNA expression. However, miR-182 expression is lower in HCV liver samples and HCV infected cells. MiR-182 reduces NKG2D ligand ULBP2 level in target cells and increases the viral load. This reveals the paradoxical role of miR-182 in NK cells and their host target hepatocytes during HCV infection [40]. Among the NK cells from patients with hepatocellular carcinoma (HCC) and healthy controls, miR-182 and NKG2D mRNA were reportedly significantly upregulated. MiR-182 is positively correlated with NKG2D mRNA expression in HCC. The overexpression of miR-182 increased NKG2D mRNA expression and ultimately enhanced NK cell activation and lytic activity in HCC [41].

MiR-29a* and miR-155 are highly expressed in NK cells from HCV infected patients, and their overexpression decreases NKG2D mRNA expression. The expression of miR-29a in NK cells of patients with HCV may inhibit NKG2D, decreasing NK cell lysis function and increasing the viral load by 80% [42].

Research shows that MICA is regulated by miRNAs in various cancers in which MICA and miRNAs often show aberrant expressions and contribute to the tumour proliferation, apoptosis, differentiation, invasion, and metastasis (Figs. 2 and 3). Human cytomegalovirus (HCMV) belongs to the family Herpesviridae, in the subfamily Betaherpesvirinae that might be exploited during immune evasion. Hcmv-miR-UL112 is reportedly encoded by HCMV and has binding sites target genes [43]. Interestingly, luciferase reporter assays in Hela cells suggested direct binding of hcmv-miR-UL112 to the 3’-UTR of MICA. However, transduction with hcmv-miR-UL112 revealed little or no reduction in MICA expression. Hcmv-miR-UL112 may also contain binding sites for human cellular miRNAs to regulate MICA expression under certain conditions. These cellular miRNAs are often aberrantly expressed in human tumour tissues and can inhibit MICA expression, which promotes tumour proliferation, apoptosis, differentiation, invasion, and metastasis, and can also aid in the avoidance of immune cell attack. The 3’-UTR region of MICA affects the protein expression. A large group of human cellular miRNAs that also bind to hcmv-miR-UL112, including miR-20a, miR-93, miR-106b, miR-302d, miR-372, miR-373, and miR-520d, could target the MICA 3’-UTR. These miRNAs specifically downregulated MICA mRNA and protein expression, showing modest effects except for miR-302d (which had no effect) and miR-372 in HeLa and DU145 cells. In primary human foreskin fibroblast (HFF) cells and human umbilical vein endothelial cells (HUVECs), the inhibition of miRNAs by antagonists resulted in higher MICA expression and enhanced killing of NK cells, due to the recognition of NKG2D, as it could be blocked by NKG2D antibodies. In contrast, the overexpression of miR-17-5p, miR-20a, miR-93, miR-106b, miR-373 and miR-520d downregulated MICA and decreased the susceptibility of NK cells due to avoid NKG2D-mediated MICA immune recognition in vitro and in vivo [43].

MiR-106b, miR-302b, miR-372 and miR-520b target the MICA 3’-UTR region and dramatically downregulate MICA surface protein expression [44]. Furthermore, miR-520b also targets the MICA promoter. MiR-520b in particular was induced by interferon-γ (IFN-γ), which could be sufficient to reduce the MICA protein expression and transcription levels in MelJuSo melanoma and HeLa cell lines [44].

Interestingly, IFN-γ enhances the expression of MICA mRNA and protein levels in human corneal epithelial cells (HCECs) by down-regulating miRNA-4448, thereby enhancing cytotoxicity and HCEC apoptosis mediated by NK and CD8^+^ T cells on the corneal epithelium [45, 46]. These findings provide novel insights into the pathogenesis of allograft rejection [47].

Papillary thyroid carcinoma (PTC) is associated with intratumoural and lymphocytic infiltration, whereas tumour-infiltrating lymphocytes and background lymphocytic thyroiditis (LT) are associated with thyroid cancer [48]. MiR-146b-5p was significantly downregulated in PTC-LT cells [49]. MICA and NKG2D are significantly overexpressed in PTC-LT and inversely correlated with miR-146b-5p expression. There is a potential binding site for miR-146b-5p in the 3’-UTR of MICA [50]. The downregulation of miR-146b-5p increased MICA and NKG2D expression in PTC and cancer cells, respectively. MICA was only detected in PTC-LT cases in the cytoplasm of tumour cells or between follicles as a secreted protein; however, MICA staining was not observed in PTC-no LT on immunofluorescence staining or confocal microscopy. NKG2D was detected in only a small number of tumour-infiltrating lymphocytes. Inhibition of the MICA-NKG2D axis by miR-146b-5p may be one way in which PTC cells help tumours evade the immune response [50].

Nasopharyngeal carcinoma (NPC) has an epithelial cell origin. Exposure to EBV is a common factor implicated in NPC aetiology [51]. In NPC, the cytotoxic activity of NK cells in NPC patients was reduced in compared to that in normal individuals [52]. The alternatively spliced transcripts are called the Bam HI A rightward transcripts (BARTs) are expressed at high levels endogenously in NPC, while EBV-encoded miRNA BART7 (EBV-miR-BART7) is a functional TGF-β1 inhibitor. MICA mRNA expression was upregulated in the HK1 NPC cell line and increased remarkably in response to TGF-β1 treatment. In NPC cells, EBV-miR-BART7 inhibits TGF-β1/c-Myc, leading to a significant decrease in their expression. Moreover, EBV-miR-BART7 suppresses MICA mRNA and protein expression, reducing the sensitivity of NPC cells to NK92MI cells [53].

Multiple myeloma is an aggressive cancer characterized by the clonal expansion of cancerous plasma cells in the bone marrow, a phenomenon that is supported by the progressive impairment of immune surveillance, primarily due to alterations in T lymphocytes and NK cells [54]. One study showed that specific small molecule inhibitors of the BET family of bromodomains (BETi) enhanced MICA promoter activity and upregulated MICA protein and mRNA levels, increasing their susceptibility to NK cell recognition and killing [55]. BETi reduced c-Myc and IRF4 expressions and upregulated microRNA-125b-5p (miR-125b) expression. Moreover, miR-125b as a tumour suppressor is commonly deregulated in cancer, while its overexpression downregulates c-Myc and IRF4 mRNA expression but upregulates MICA mRNA and protein expression [56]. Thus, the transcriptional repressor c-Myc of MICA and IRF4 is an inhibitor of MICA promoter activity in multiple myeloma cells [57].

Colorectal cancer (CRC) is among the most common fatal cancers worldwide [58, 59]. The miR-20a expression level was significantly increased in CRC tissues [60, 61]. Ovarian cancer (OC) is the most lethal malignancy in a woman’s reproductive system, and immunotherapy has a limited impact on treatment outcomes. The miR-20a expression level was also significantly increased in OC tissues [62]. The upregulation of miR-20a in patients results in shorter overall survival and plays an important role in ovarian tumourigenesis and anti-tumour immunity. MiR-20a can directly bind to the MICA 3’-UTR and its overexpression suppresses the MICA mRNA and cell surface MICA protein levels, resulting in reduced NKG2D-mediated killing. Moreover, miR-20a expression is negatively correlated with MICA. NKG2D signalling affects miR-20a, which controls the sensitivity of CRC and OC cells to NK cells. This indicates that miR-20a downregulates MICA expression, reduces NK cell cytotoxicity, and is NKG2D-dependent in cancer cells to promote tumour growth [61, 62].

BC is one of the most common malignancies among females. MICA mRNA and protein expression levels are lower in normal breast tissues than in paired BC tissues [63]. The MICA of BC cells at early stages (I and II) was significantly higher than that of advanced (stage III) BC cells. Members of the miR-17-92 cluster, including miR-20a, miR-20b, miR-93, and miR-106b reportedly down-regulate MICA [64]. Hence, the silencing of MICA-targeting miRNAs contributes to immune recognition in vivo. Histone deacetylase inhibitors (HDACi) are commonly used in clinical trials to treat malignant tumours in humans. In particular, suberoylanilide hydroxamic acid (SAHA) and valproic acid (VPA) are vital HDACi that have been used in clinical trials to treat cutaneous T-cell lymphoma and may induce cancer cell differentiation and apoptosis [65]. SAHA and VPA treatment upregulates MICA expression and decreases miR-17-92 cluster expression, thereby increasing the sensitivity of NK cell mediated cytotoxicity. Moreover, miR-519a-3p expression is upregulated in BC cells and associated with poor survival. MiR-519a-3p binds to the MICA 3’-UTR and reduces the MICA mRNA levels and surface protein levels. MiR-519a-3p impairs NK cell-mediated killing of BC cells and deregulates apoptosis by downregulating MICA [66].

The most aggressive subtype of BC, triple-negative breast cancer (TNBC), has the highest recurrence rates due to distant metastases and poor prognosis [63]. MiR-20a was reportedly specifically overexpressed in TNBC. According to one study, 3’-O-acetylvitexin affected cytotoxic activity and successfully increased MICA expression, which impacted miR-20a validated targets [67]. Another study showed that MICA expression was significantly increased as downstream targets of the MALAT1/p53/miR-155/miR-146a circuit affected by methoxylated quercetin glycoside (MQG), which alters the immunogenic and oncogenic profiles of BC [68]. It was essential to investigate the impact of MQG on miR-155 and miR-146a targets of MICA, known to be markedly downregulated in MDA MB-231 cells, and vital immune-inhibitory cytokines (tumour necrosis factor–α, interleukin-10), which are known to play a dominant role in potentiation of the immune suppressive microenvironment in BC patients [68].

HCC is the third most common cause of cancer-related death worldwide [69]. MICA protein expression levels showed different statuses in four representative HCC cell lines, including those from the same organ. According to flow cytometry and immunohistochemistry, Hep3B and PLC/PRF/5 cells express substantial MICA protein levels, while Huh7 and HLE cells do not express MICA protein [70]. Thus, miR-93 and miR-106b located on the miR-25-93-106b cluster, are considered to target MICA 3’-UTR. MICA protein expression is suppressed by miR-93 and miR-106b in HeLa and Hep3B cells. To determine the ability of MICA to bind to NKG2D, MICA and NKG2D protein expression levels were decreased by miR25-93-106b cluster overexpression, whereas their protein expression levels were increased by silencing the miR-25-93-106b cluster. The miR-25-93-106b cluster can regulate tumour progression and invasion by regulating MICA expression, while tumours are sensitive to NK cells. Regulating the MICA protein expression is essential for preventing the development of HCC during chronic hepatitis viral infection [70].

SAHA treatment dramatically increased the susceptibility of HepG2 and H7402 cells to cytolysis by interleukin-2-activated NK cells and upregulated MICA protein expression. The miR-17-92 cluster (miR-20a, miR-93, and miR-106b) targeted MICA and increased MICA surface protein levels, and the miRNAs were downregulated by SAHA treatment. These results suggested that HDACi promotes MICA protein expression by inhibiting the transcription of miRNAs targeting MICA in HCC [71].

Lung cancer is the leading cause of cancer related death in men and women worldwide [72]. When detected in human non-small cell lung carcinoma cell lines, squamous cell carcinoma tissues, and adenocarcinoma compared to lung epithelial cells and adjacent normal lung tissues, MICA transcript or protein expression was low or absent, and miR-183 expression was higher. Thus, after bioinformatics analysis and luciferase reporter analyses, miR-183 targeted to the MICA 3’-UTR. MiR-183 expression was negatively correlated with MICA expression. Antisense miR-183 transfection successfully suppressed miR-183 expression and upregulated MICA protein expression in H1355 and H1299 lung tumour cells. TGF-β reduced the effectiveness of NK cells and induced miR-183, leading to MICA suppression, and contributing to its immune escape [73]. The knockdown of TGF-β mRNA effectively resulted in reduced miR-183 expression and upregulated MICA protein expression. The silencing of miR-183 and overexpression of MICA contributed to tumour cells lysis by activating CD8^+^ T cells via the MICA-NKG2D pathway [74].

**Fig. 4:**
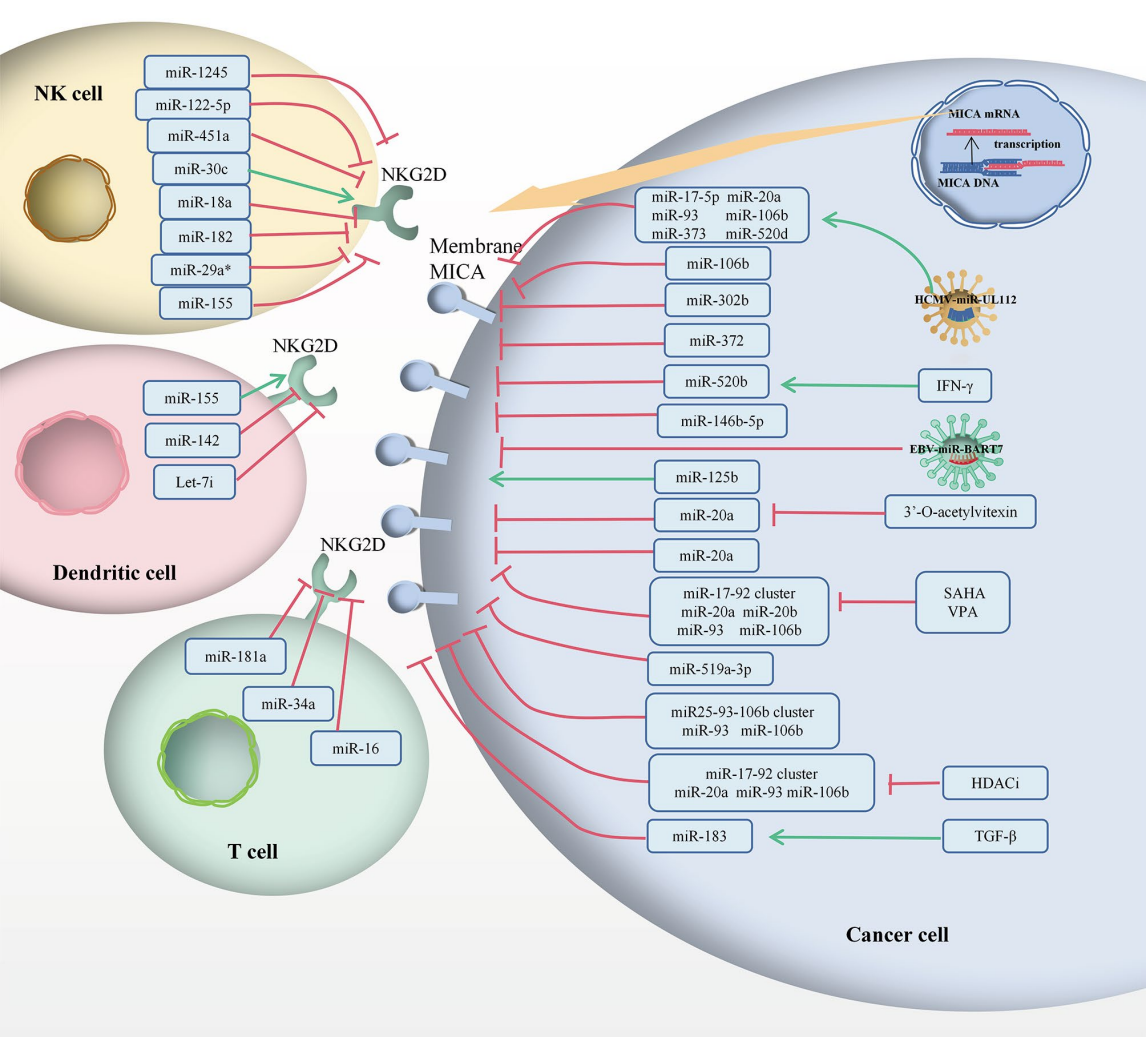
The MICA/NKG2D pathway was regulated by human miRNAs to induce the immune response. The green arrow indicates stimulatory modification, while the red “T” symbol indicates inhibitory modification

According to the Sankey diagram (Fig. 3), MICA and NKG2D can be targeted by a variety of common ncRNAs. For example, the top five ncRNAs were miR-20a, miR-106b, miR-93, miR-155 and miR-372, indicating that different tumours mediate the role of MICA/NKG2D in immunoregulation through the same specific miRNA. Particularly, the regulation of MICA by miR-20a in BC is similar to that in OC, CRC and HCC. It is possible that even ncRNAs from different tumours could lead to the same regulatory pathway in targeting immune cells, acting as powerful regulators of immunomodulatory genes involved in tumour immunity, mediating immune escape, highlighting their potential use as biomarkers, and manipulating ncRNA expression as therapeutic interventions (Table 1; Fig. 4).

### Role of non-coding RNA in regulation of MICA/ NKG2D axis in different human cancers

**Fig. 5:**
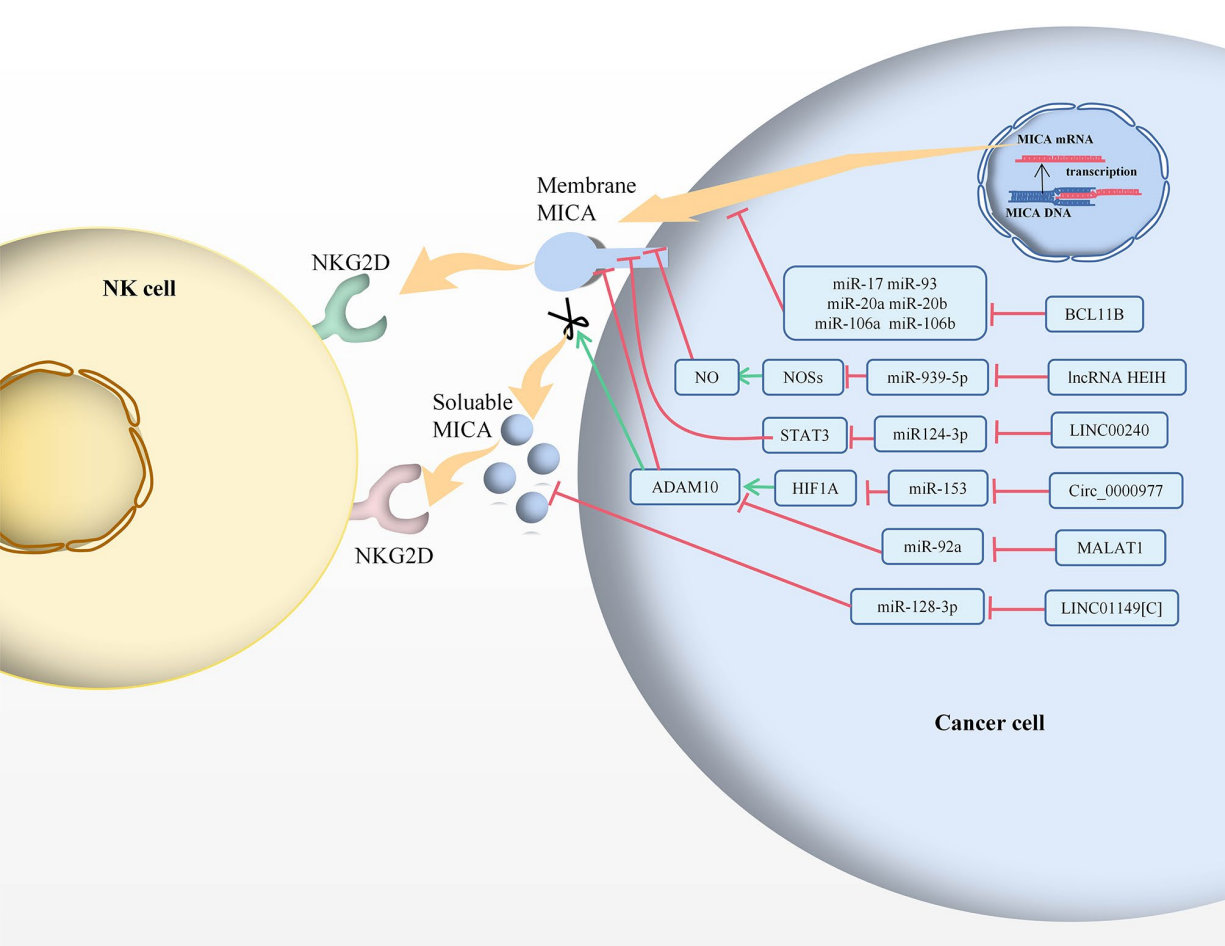
MICA mediated the immune response of cancer cells from the perspective of circRNAs or the lncRNAs-miRNAs-mRNAs axis. The green arrow indicates stimulatory modification, while the red “T” symbol indicates inhibitory modification. The green NKG2D indicates a stimulatory immune response, while the red NKG2D indicates an inhibitory immune response

CeRNAs suppress intracellular miRNA function and regulate mRNA expression via shared miRNA response elements (MREs) [75]. Our review focuses on MICA-related ceRNA networks, which are proven powerful tools for deciphering cancer mechanisms and predicting therapeutic responses at the system level (Table 2; Figs. 2, 3 and 5). BCL11B is considered a ceRNA sponge that binds to the miRNAs that positively regulate MICA protein expression [75]. MiR-17, miR-93, miR-20a, miR-20b, miR-106a, and miR-106b have binding sites with the MICA 3’-UTR and the BCL11B 3’-UTR. When miR-17, miR-93, miR-20a, miR-20b, miR-106a, and miR-106b mimics were transfected into HCT116 cells, the MICA and BCL11B protein expression levels were significantly downregulated. These results suggest that mature miRNAs negatively regulate MICA and BCL11B. BCL11B knockdown leads to a decrease in MICA, which then leads to a decrease in the lytic sensitivity of NK cells and influences in NK cells cytotoxicity [75]. Therefore, the MICA-NKG2D pathway mediates an immune response against the killing effect of NK cells during tumourigenesis.

**Table 2:** CeRNA networks regulated MICA in cancers

Cancer type	Competing endogenous RNAs	Cancer tissues/cells status	Sponge miRNAs	Cancer tissues/cells status	Competitor mRNAs	Cancer tissues/cells status	CeRNA Network	CeRNA Role	Functions	References
Colon cancer	BCL11B	Downregulated	miR-17, miR-93, miR-20a, miR-20b, miR-106a, miR-106b	Upregulated	MICA	Downregulated	BCL11B/ miR-17, miR-93, miR-20a, miR-20b, miR-106a, miR-106b/MICA	Tumour suppressorgene	MiRNAs negatively regulate MICA and BCL11B. MICA-NKG2D pathway mediates the immune response to the killing effect of NK cells in tumourigenesis.	[75]
Triple-negative breast cancer	LncRNA HEIH	Upregulated	miR-939-5p	Downregulated	NOSs/NO/MICA	Downregulated/Downregulated/Upregulated	LncRNA HEIH/miR-939-5p/NOSs/NO/MICA	Oncogene	Silencing of lncRNA HEIH causes a significant down-regulation of NOSs, NO production, cellular viability, migration ability and increases the expression of MICA in TNBC cells.	[78]
Hepatocelluar carcinoma	LINC01149[C]	Upregulated	miR-128-3p	Downregulated	MICA	Upregulated	LINC01149[C]/miR-128-3p/MICA	Oncogene	LINC01149 genotypes are associated with sMICA level in HCC patients and the C allele contributed to higher sMICA level and increase risk of hepatocelllular carcinoma in persistent HBV infection and HCC.	[18]
Pancreatic cancer	Circ_0000977	Upregulated	miR-153	Downregulated	HIF1A/ADAM10	Upregulated/Upregulated	Circ_0000977/miR-153/HIF1A/ADAM10/MICA	Oncogene	Circ_0000977 could inhibit the expression of miR-153 and reverse the downregulation of HIF1A and ADAM10 mediated by miR-153, circ_0000977 upregulates ADAM10 to shedding of mMICA transforming to sMICA; hyporesponsiveness in NK cells.	[85]
Neuroblastoma	MALAT1	Upregulated	miR-92a	Downregulated	ADAM10	Upregulated	MALAT1/miR-92a/ADAM10/MICA	Oncogene	MALAT1 shedding of mMICA transforming to sMICA contributes to the formation of a suppressive immune microenvironment and promoting immune escape.	[88]
Cervical cancer	LINC00240	Upregulated	miR-124-3p	Downregulated	STAT3/MICA	Upregulated/Downregulated	LINC00240/miR124-3p/STAT3/MICA	Oncogene	LINC00240 significantly downregulates miR-124-3p, and decreases MICA mRNA and protein expression and reduced the cytotoxic activity of NKT cells through regulation of STAT3 promoted cervical cancer progression in vitro and vivo.	[91]

Previous reports demonstrate that lncRNA hepatocellular carcinoma up-regulated EZH2-associated (HEIH) is an oncogene because of its high expression levels in TNBC [76, 77]. The lncRNA HEIH 3’-UTR binds to miR-939-5p [76, 77]. In contrast, miR-939-5p is dramatically downregulated in BC, luminal B, and TNBC tissues [78]. Nitric oxide (NO) plays an important role in tumourgenesis and immune responses, and the NO-induced by NO synthase (NOS) regulates immunity [79]. Thus, the efficient transfection of miR-939-5p in MDA-MB-231 cells resulted in low expression of NOS2 and NOS3 transcripts as well as NO production [79]. In contrast, the overexpression of miR-939-5p decreased lncRNA HEIH, whereas the silencing of lncRNA HEIH significantly downregulated NOS2, NO production, cellular viability, and migration ability and increased MICA and miR-939-5p expression in TNBC cells. Hence, the lncRNA HEIH and miR-939-5p may impact MICA alterations. Mechanistically, it was a hypothesised that the lncRNA HEIH/miR-939-5p mediates the NOSs/NO/MICA axis in TNBC [79].

Few functional studies have examined the variations in lncRNAs. Interpreting disease-causing variations, particularly in long non-coding regions, has become one of the most difficult tasks, indicating the potential relevance of functional lncRNAs variants in complicated disorders [18]. HBV infection is among the most common viral infections worldwide, and chronic hepatitis B (CHB) may further increase the prevalence of HCC [80].

LINC01149 has two variants: rs8044472 and rs2844512. The LINC01149 rs28445512 [C] allele significantly decreases the risk of persistent HBV infection but increases the risk of HCC, which is associated with persistent HBV infection and HCC susceptibility [18]. The LINC01149 rs2844512 [C] variant has binding sites targets miR-128-3p and is downregulated by miR-128-3p. Moreover, miR-128-3p expression levels in adjacent normal tissues were significantly higher than those in HCC tissues in TCGA. MiR-128-3p is a target of MICA [18]. The MICA mRNA expression levels in HCC tissues were significantly higher than those in adjacent normal tissues from TCGA and were decreased by miR-128-3p. Thus, LINC01149 [C] acts as a sponge ceRNA that induces MICA expression by bind to miR-128-3p. The LINC01149 genotypes are associated with sMICA levels in patients with HCC and the C allele contributes to higher sMICA levels. LINC01149 [C] contributes to increased sMICA released via proteolytic shedding, NK cells exhaustion, and tumour immune evasion. The regulatory highlights indicate that the LINC01149[C]/miR-128-3p/MICA axis facilitates spontaneous HBV recovery and increases the risk of HCC in persistent HBV infection and HCC [18].

A few malignant cells exist inside the intratumoural hypoxic microenvironment of extracellular matrix, fibroblasts, endothelial cells, and immune cells in pancreatic cancer (PC) [81]. Hypoxia typically causes the release of immunosuppressive chemicals in tumour cells and promotes cancer cell resistance to cytotoxic T-cell-mediated lysis [82]. Hypoxia inducible factor 1 alpha (HIF1A) is significantly upregulated in PC tissues [82]. Hypoxia is considered an important inducer of tumour cell resistance to immune-effector mediated cleavage, which occurs with increased ADAM10 expression via HIF1A dependent pathways. ADAM10 and mMICA expression were significantly upregulated and downregulated in the high HIF1A expression group from the peripheral blood mononuclear cells (PBMCs) of patients, while serum sMICA levels were increased [83]. Moreover, hypoxia could significantly upregulate sMICA content in the culture supernatant, whereas HIF1A inhibition could increase the mMICA levels and numbers of NKG2D positive NK cells, decrease sMICA levels, and enhance the killing effect of NK cells on Panc-1 cells [84]. Thus, ADAM10 is negatively correlated with mMICA expression [13]. In the HIF1A high-expression group, circ_0000977 expression was the highest and positively correlated with ADAM10, but negatively correlated with mMICA. Similar to HIF1A and ADAM10, circ_0000977 expression was induced by hypoxia in a concentration-dependent manner in Panc-1 cells, significantly increasing sMICA level in the supernatant, suggesting that circ 0000977, HIF1A, and ADAM10 expressions are all highly increased in PC tissues [85]. Moreover, miR-153 was most significantly downregulated in PC tissues of the HIF1A high-expression group and negatively correlated with ADAM10 expression and circ_0000977 expressions. MiR-153 directly binds to circ_0000977, HIF1A, and ADAM10. In addition, circ_0000977 could inhibit miR-153 expression and reverse the downregulation of HIF1A and ADAM10 dysregulation mediated by miR-153. Notably, circ_0000977 as a sponge (ceRNA) competitively binds to miR-153 to eliminate miR-153-mediated inhibition of HIF1A and ADAM10 [85]. Increased ADAM10 expression is responsible for the enhanced shedding of mMICA on the surface of pancreatic ductal adenocarcinoma (PDAC) cells, which is converted to sMICA resulting in reduced reactivity of NK cells due to the degradation of NKG2D, which is an important aspect of immune escape [84]. CircRNAs and miRNAs show some crucial biological properties that are closely related to their functions and clinical implications, and dysregulated in PC [85, 86].

Neuroblastoma is among the most common extracranial malignant solid tumours of childhood. Its clinical symptoms vary widely among children, with some tumours regressing on their own [87]. After low-dose chemotherapeutic drug stimulation induced cellular senescence of neuroblastoma cell lines, MICA release was significantly increased in both membranes and exosomes [87]. ADAM10 mRNA and protein levels are upregulated in senescent cells, possibly related to MICA secretion. Chemotherapy combined with ADAM10 inhibition reduces MICA released from senescent cells and inhibits immune escape. In contrast, the downregulation of NKG2D expression was significantly inhibited by exosomal MICA blockage. MiR-92a-3p has common binding sites with MALAT1, and ADAM10. This result also suggested that MALAT1 expression was upregulated, and that miR-92a-3p was downregulated in IMR-32 cells. These findings suggest that MALAT1 upregulates ADAM10 expression by acting as a ceRNA and the competitively inhibiting miR-92a-3p, which affects on ADAM10 expression by promoting sMICA mediated immune escape. MALAT1 knockdown in senescent cells downregulated ADAM10 expression, which inhibited MICA released and suppressed immune escape. This finding provides new insights into the immune escape mechanism of senescent cells in that MALAT1 (ceRNA) promotes the shedding of MICA through the MALAT1/miR-92a/ADAM10 axis, contributing to the formation of a suppressive immune microenvironment and promoting immune evasion [88].

Cervical cancer is the second most lethal gynaecological malignancy worldwide [63]. NcRNAs are crucial gene masters and potential markers of cervical cancer [26, 89]. LINC00240 expression was reportedly increased in cervical cancer tissues and in the cytoplasm compared to adjacent non-tumour tissues as well as in the nuclei of HeLa and C-33 A cells. High LINC00240 levels resulted in significantly shorter overall-survival. Otherwise, miR-124-3p expression is dramatically decreased in cervical cancer tissues. LINC00240 targeted to miR-124-3p induced cervical cancer cell proliferation, migration, and invasion. A negative correlation was observed between LINC00240 and miR-124-3p levels. A recognized transcription factor called STAT3 has been linked to tumours development [90]. Increased STAT3 expression was noted in cervical cancer tissue. A positive correlation was observed between LINC00240 and STAT3 expressions. STAT3 directly binds to miR-124-3p and directly interacts with the MICA promoter [91]. MICA plays a key role in NKT cell activation and serves as a downstream target of STAT3. STAT3 from tumour cells can reduce anti-tumour immunity by suppressing MICA and generating pro-inflammatory chemokines. Thus, LINC00240 significantly downregulates miR-124-3p, decreases MICA mRNA and protein expressions and reduces the cytotoxic activity of NKT cells by regulating STAT3 promoted cervical cancer progression in vitro and in vivo. LINC00240 acts as a sponge by downregulating miR-124-3p and inducing STAT3 expression to decrease MICA expression. The cytotoxic activity of NKT cells is facilitated by the LINC00240/miR124-3p/STAT3/MICA axis in cervical cancer [91].

## Conclusions and further perspectives

The recent emergence of immune checkpoint therapy has become the latest approach in the fight against cancer, including research on programmed death 1 (PD-1) and programmed death ligand 1 (PD-L1), the discoverers of which were awarded the 2018 Nobel Prize in Physiology or Medicine [92]. For example, there are potential binding sites of miR155 with the 3’-UTR of PD-L1. MiR155 modulates the PD-1/PD-L1 mediated interaction between B lymphoma cells and CD8^+^ T cells via the regulation of PD-L1 expression [93]. Additionally, high levels of circRNA fibroblast growth factor receptor 1 (circFGFR1) are associated with cytotoxic T lymphocyte exclusion and resistance to anti-PD-1 therapy in lung cancer cells that targets miR-381-3p binding to the target gene C-X-C motif chemokine receptor 4 (CXCR4) [94]. CircFGFR1 inhibits miR-381-3p by sponging, which mediates the resistance of lung cancer cells to PD-1 blockers [95].

In summary, miRNAs are small ncRNAs that bind to complementary or partially complementary sites in the 3’-UTR of target genes to inhibit target transcript protein translation and induce mRNA degradation or cleavage of ligands such as NKG2D and MICA. MICA is a double-edged sword that acts as target molecules for NK cell immune surveillance and as mediators of tumour immune escape. The MICA/NKG2D pathway is dysregulated by miRNAs, such as HCMV-miR-UL112, EBV-miR-BART7 and cellular miRNAs (Table 1) (Figs. 2 and 3). This insight has important implications for understanding disease progression and molecular targeting [96]. MiRNAs that regulate the transport or downstream targets of NKG2D and MICA may also have a significantly impact the MICA-NKG2D immune signalling pathway (Fig. 4). Currently, little is known about the correlation between these ceRNAs and ncRNAs, the prognostic roles of MICA, or the therapeutic effects of the MICA-NKG2D antibodies. Significant advances have been made in the use of miRNAs as diagnostic targets [34]. Therefore, it is essential to determine whether additional circRNAs, lncRNAs, and miRNAs can serve as biomarkers and therapeutic targets (Table 2; Fig. 5). The corresponding ncRNA drugs are among the most powerful alternatives to tumour drug therapies, specifically since they target the tumour current state and enable its precise regulation. It is possible to further improve the therapeutic success of immune checkpoint inhibitors combined with ncRNA mimics or inhibitors. Therefore, drugs can be designed to target ncRNAs and genes closely related to these tumours, further advancing personalised precision therapies. In addition to the development of mechanisms, another challenge for clinical applications is the design of inhibitors that regulate harmful ncRNAs. These ncRNAs are also potentially useful biomarkers for determining the prognosis of MICA-NKG2D antibody treatment. It is possible that in addition to further understanding, the molecular regulatory network of MICA-NKG2D is being targeted for cancer treatment, and an improved understanding of its post-transcriptional regulation may open new avenues for next-generation immunotherapies.

## Data Availability

Figure 2 were retrieved from Figdraw (https://www.figdraw.com/static/index.html ID:SYSUObfb6b).

## Abbreviations

3’-UTR: 3’-untranslated regions
ACC: Adrenocortical
ADAMs: A Disintegrin Metalloproteinase Domains
BARTs: Bam HI A rightward transcripts
BC: Breast cancer
BETi: BET family of bromodomains
BLCA: Bladder Cancer
BRCA: Breast Cancer
ceRNA: Competitive endogenous RNA
CESC: Cervical Cancer
CHB: Chronic hepatitis B
CHOL: Bile Duct Cancer
circFGFR1: circRNA fibroblast growth factor receptor 1
circRNA: Circular RNA
COAD: Colon Cancer
COVID-19: Coronavirus disease 2019
CRC: Colorectal cancer
CXCR4: C-X-C motif chemokine receptor 4
DLBC: Lymphoid Neoplasm Diffuse Large B-cell Lymphoma
EBV: Epstein-Barr virus
ESCA: Esophageal carcinoma
GBM: Glioblastoma multiforme
HBV: Hepatitis B virus
HCC: Hepatocellular carcinoma
HCECs: Human corneal epithelial cells
HCMV: Human cytomegalovirus
HDACi: Histone deacetylase inhibitors
HFF: Human foreskin fibroblast
HIF1A: Hypoxia inducible factor 1 alpha
HNSC: Head and Neck squamous cell carcinoma
HUVECs: Human umbilical vein endothelial cells
IDO1: Indoleamine-2, 3-dioxygenase 1
KICH: Kidney Chromophobe
KIRC: Kidney renal clear cell carcinoma
KIRP: Kidney renal papillary cell carcinoma
KLRK1: Killer cell lectin like receptor K1
LAML: Acute Myeloid Leukemia
LGG: Brain Lower Grade Glioma
LIHC: Liver hepatocellular carcinoma
lncRNA: Long noncoding RNA
lncRNA: HEIH Hepatocellular carcinoma upregulated EZH2-associated lncRNA
LT: Lymphocytic thyroiditis
LUAD: Lung adenocarcinoma
LUSC: Lung squamous cell carcinoma
MESO: Mesothelioma
MICA: Major histocompatibility complex class I related chain A
miRNA: microRNA
mMICA: Membrane-bound major histocompatibility complex class I related chain A
MMPs: Matrix metalloproteinases
MQG: Methoxylated quercitin glycoside
MREs: miRNA response elements
ncRNAs: Noncoding RNAs
NK: Cells Natural killer cells
NKG2D: Natural-killer group 2 member D receptor
NKG2DLs: Natural-killer group 2 member D receptor ligands
NO: Nitric oxide
NPC: Nasopharyngeal carcinoma
NSCLC: Nonsmall cell lung carcinoma
OC: Ovarian cancer
OV: Ovarian serous cystadenocarcinoma
PAAD: Pancreatic adenocarcinoma
PC: Pancreatic cancer
PCPG: Pheochromocytoma and Paraganglioma
PD-1: Programmed death 1
PD-L1: Programmed death ligand 1
PRAD: Prostate adenocarcinoma
PTC: Papillary thyroid carcinoma
READ: Rectum adenocarcinoma
RAET: Retinoic acid early transcripts
SAHA: Suberoylanilide hydroxamic acid
SARC: Sarcoma
SARS-CoV-2: severe acute respiratory syndrome coronavirus 2
SKCM: Skin Cutaneous Melanoma
sMICA: Soluble major histocompatibility complex class I related chain A STAD Stomach adenocarcinoma
TGCT: Testicular Germ Cell tumours
TGF-β1: Transforming growth factor–β1
THCA: Thyroid carcinoma
THYM: Thymoma
TNBC: Triple negative breast cancer
UCEC: Uterine Corpus Endometrial Carcinoma
UCS: Uterine Carcinosarcoma
UVM: Uveal Melanoma
UL-16: Binding protein (ULBP) family
VPA: Valproic acid
